# Danqi Pill regulates lipid metabolism disorder induced by myocardial ischemia through FATP-CPTI pathway

**DOI:** 10.1186/s12906-015-0548-0

**Published:** 2015-02-21

**Authors:** Yong Wang, Chun Li, Qiyan Wang, Tianjiao Shi, Jing Wang, Hui Chen, Yan Wu, Jing Han, Shuzhen Guo, Yuanyuan Wang, Wei Wang

**Affiliations:** Beijing University of Chinese Medicine, Beijing, 100029 China; Modern Research Center for Traditional Chinese Medicine, Beijing University of Chinese Medicine, Beijing, 100029 China

**Keywords:** Lipid metabolism disorder, Myocardial ischemia, FATP-CPTI pathway

## Abstract

**Background:**

Danqi Pill (DQP), which contains Chinese herbs *Salvia miltiorrhiza Bunge and Panax notoginseng,* is widely used in the treatment of myocardial ischemia (MI) in China. Its regulatory effects on MI-associated lipid metabolism disorders haven’t been comprehensively studied so far. We aimed to systematically investigate the regulatory mechanism of DQP on myocardial ischemia-induced lipid metabolism disorders.

**Methods:**

Myocardial ischemia rat model was induced by left anterior descending coronary artery ligation. The rat models were divided into three groups: model group with administration of normal saline, study group with administration of DanQi aqueous solution (1.5 mg/kg) and positive-control group with administration of pravastatin aqueous solution (1.2 mg/kg). In addition, another sham-operated group was set as negative control. At 28 days after treatment, cardiac function and degree of lipid metabolism disorders in rats of different groups were measured.

**Results:**

Plasma lipid disorders were induced by myocardial ischemia, with manifestation of up-regulation of triglyceride (TG), low density lipoprotein (LDL), Apolipoprotein B (Apo-B) and 3-hydroxy-3-methyl glutaryl coenzyme A reductase (HMGCR). DQP could down-regulate the levels of TG, LDL, Apo-B and HMGCR. The Lipid transport pathway, fatty acids transport protein (FATP) and Carnitine palmitoyltransferase I (CPTI) were down-regulated in model group. DQP could improve plasma lipid metabolism by up-regulating this lipid transport pathway. The transcription factors peroxisome proliferator-activated receptor α (PPARα) and retinoid X receptors (RXRs), which regulate lipid metabolism, were also up-regulated by DQP. Furthermore, DQP was able to improve heart function and up-regulate ejection fraction (EF) by increasing the cardiac diastolic volume.

**Conclusions:**

Our study reveals that DQP would be an ideal alternative drug for the treatment of dyslipidemia which is induced by myocardial ischemia.

## Background

Coronary heart disease (CHD) is one of the major causes of death worldwide [1,2]. CHD is the progress of the coronary arteries stenosis, usually caused by atherosclerosis, which is the buildup of cholesterol and fatty deposits on the inner walls of the arteries [3]. The plaque formed in the artery could restrict blood flow to the heart muscles and cause myocardial ischemia. Lipid peroxidation induced by lipid infiltration is considered to be the main pathological mechanism of myocardial ischemia [4]. Some novel drugs targeting lipid metabolism have been developed to treat or reduce the risk of myocardial ischemia caused by CHD [5]. Statins, which are HMG-CoA reductase inhibitors, are widely used for their cholesterol-lowering properties and have been proven to be able to reduce cardiovascular disease risk [6]. Statins mainly reduce plasma levels of LDL, while having little effect on TG [7]. Since 1970s, a number of herbal compounds have been developed to treat MI. Among them, Danshen and Sanqi are the most frequently prescribed [8-10]. Because they have definitive curative effect and conform to pharmacopoeia standard of quality control (Ministry of Health of the People’s Republic of China), Danqi Pill (DQP), which is composed of DanShen and Sanqi, was listed in Chinese Pharmacopoeia 2010 as routine drug in the clinical treatment of myocardial ischemia and impaired cardiac function [11]. Danqi could also improve microcirculation by exerting anti-platelet aggregation effect [12]. However, the effect of DQP on lipid metabolism signaling pathway hasn’t been studied comprehensively so far. Our previous study demonstrated that DQP could improve heart function, partly via its regulation of ox-LDL and arachidonic acid metabolism [13]. In this study, we aim to investigate if DQP could regulate lipid metabolism and prevent fat deposition in artery, thus intervening the clinical course of MI in CHD.

## Methods

### CHD model preparation and animal grouping

A total of 80 pathogen-free male Sprague–Dawley (SD) rats, weighing 220 ± 10 g, were selected and divided randomly into sham-operated, model, DQP treatment and Statin positive control group with 20 rats in each group. The rats were purchased from Beijing Vital River Laboratory Animal Tchnology Co., Ltd. This study was carried out in accordance with the China Physiological Society’s “Guiding Principles in the Care and Use of Animal” and approved by Animal Care Committee of Beijing University of Chinese Medicine.

Myocardial ischemia model was induced in all but the sham-operated group of rats by direct coronary ligation as previously described [14,15]. In short, SD rats were intubated and anaesthetized intraperitoneally with 1% pentobarbital sodium at the dosage of 50mg/kg. Left thoracotomy was then performed and the left anterior descending coronary artery proximal to its main branching point was ligated with a 5–0 polypropylene suture (Surgipro, CT, USA). One to two drops of lidocaine was put on the surface of the rats’ hearts immediately after ligation. SD rats in the sham-operated groups also underwent thoracotomy but their coronary artery was not ligated. Thorax was closed after thoracotomy and ligation of the left anterior descending coronary artery. Lidocaine of 0.1-0.2 ml and furosemide of 0.1-0.2 ml were injected abdominally after the thorax was closed. The rats were extubated when they could breathe sufficiently. The DQP group was treated for 28 days by daily oral gavage with dosages of 1.5 mg/kg of DQP aqueous solution (Beijing university of Chinese Medicine, Beijing, China, Series: 6128006). The positive control group was also treated for 28 days by daily oral gavage of aqueous pravastatin solution (Bristol-Myers Squibb, China, Series: H19980197) at the dosage of 1.2 mg/kg. Both the sham-operated and model groups received the same volume of saline water in the same way as the other two groups. 28 days after operation, blood samples were collected via abdominal aorta puncture after all animals were anaesthetized using pentobarbital sodium following an overnight fast. After centrifugation, plasma was collected and stored at −80 °C for further analysis. Left ventricle tissue was put into liquid nitrogen for further analysis.

The DQP used in the present study was a Chinese patent medicine, manufactured by TongrenTang (Beijing, China, Z11020471) using 2 Chinese herbs at a composition of 1:1(150 g S. miltiorrhiza bunge and 150 g P. notoginseng). It strictly fulfils the China Pharmacopoeia standard of quality control (Ministry of Health of the People’s Republic of China Pharmacopoeia Committee, 2005). A voucher specimen (Series: 6128006) was kept in the Beijing University of Chinese Medicine.

### Echocardiographic assessment of heart function

Echocardiography was applied to measure cardiac function related parameters, including ejection fraction (EF), left ventricular end-systolic diameter (LVEDs), left ventricular end-diastolic diameter (LVEDd),and other indicators including interventricular septum thickness at end-systole (IVSs), interventricular septum thickness at end-diastole (IVSd), the diastolic thickness of the LV posterior wall (LVPWd), and LV posterior wall thickness at end-systole (LVPWs). LV dimension (LVD) was measured by M model. Fractional shortening (FS%) was calculated as: FS% = [(LVEDd − LVEDs)/LVEDd] × 100%.

### Measurement of lipid metabolism indicators

The plasma was homogenized in saline which contains heparin as anticoagulant at the concentration of 20 μL/mL. The homogenate was centrifuged at 8000 × g for 10 min and supernatant was used for detection of plasma indicators. Plasma Total Cholesterol (TC), TG, high density lipoprotein (HDL) and LDL levels were measured by automatic biochemical analyzer (HITACH17080, Japan) following the instructions of the company.

Levels of Apolipoprotein A I (ApoA-I), Apolipoprotein B (Apo-B), lipoprotein (a) (Lp(a)) and HMGCR were quantified in duplicate using commercial ELISA kits (Crystal Chem Inc., Downer’s Grove, USA). Each assay was performed following their respective protocols. Standards at a series of concentrations were run in parallel with the samples and the concentrations in the samples were calculated in reference to the corresponding standard curves.

Left ventricle homogenates were prepared from rats for the analysis of protein levels. Equal amounts of protein extracts (20 mg) were separated by 12.5% or 15% sodium dodecyl sulphate (SDS)-polyacrylamide gel electrophoresis (Bio-Rad, CA, U.S.A.) and transferred to nitrocellulose membranes electrophoretically (semidry transfer). Membranes were blocked with 5% non-fat dry milk in Tris-buffered saline (20 mM Tris, pH 7.6, 137 mM NaCl) with 0.1% Tween 20, washed, and then incubated with primary antibody. Primary antibodies employed included: goat polyclonal antiglyceraldehyde-3-phosphate dehydrogenase (GAPDH) and anti-ApoAI\FATP\CPTI\PPARα\RXR\NR2C2 (Abcam., U.S.A.). The primary antibody was firstly incubated, and then the secondary antibodies (Santa Cruz Biotechnology Inc., CA, U.S.A.) was added. After exposed by chemiluminescence developing agents, the protein levels and GAPDH in each sample were evaluated. The gel was scanned and the band densities were quantified. The other indicators were normalized by the GAPDH band densities to determine their concentrations.

### Statistical analysis

ANOVA using SAS 9.2 statistical software (SAS Institute, NC, USA) was applied to evaluate between-group differences in the outcome variables, follow-up least significant differences (LSD) analysis verified these differences were significant. P < 0.05 was considered statistically significant. Results were presented as mean values with their corresponding standard deviations.

## Results

### Effects of DQP on parameters related to cardiac function

**Figure 1:**
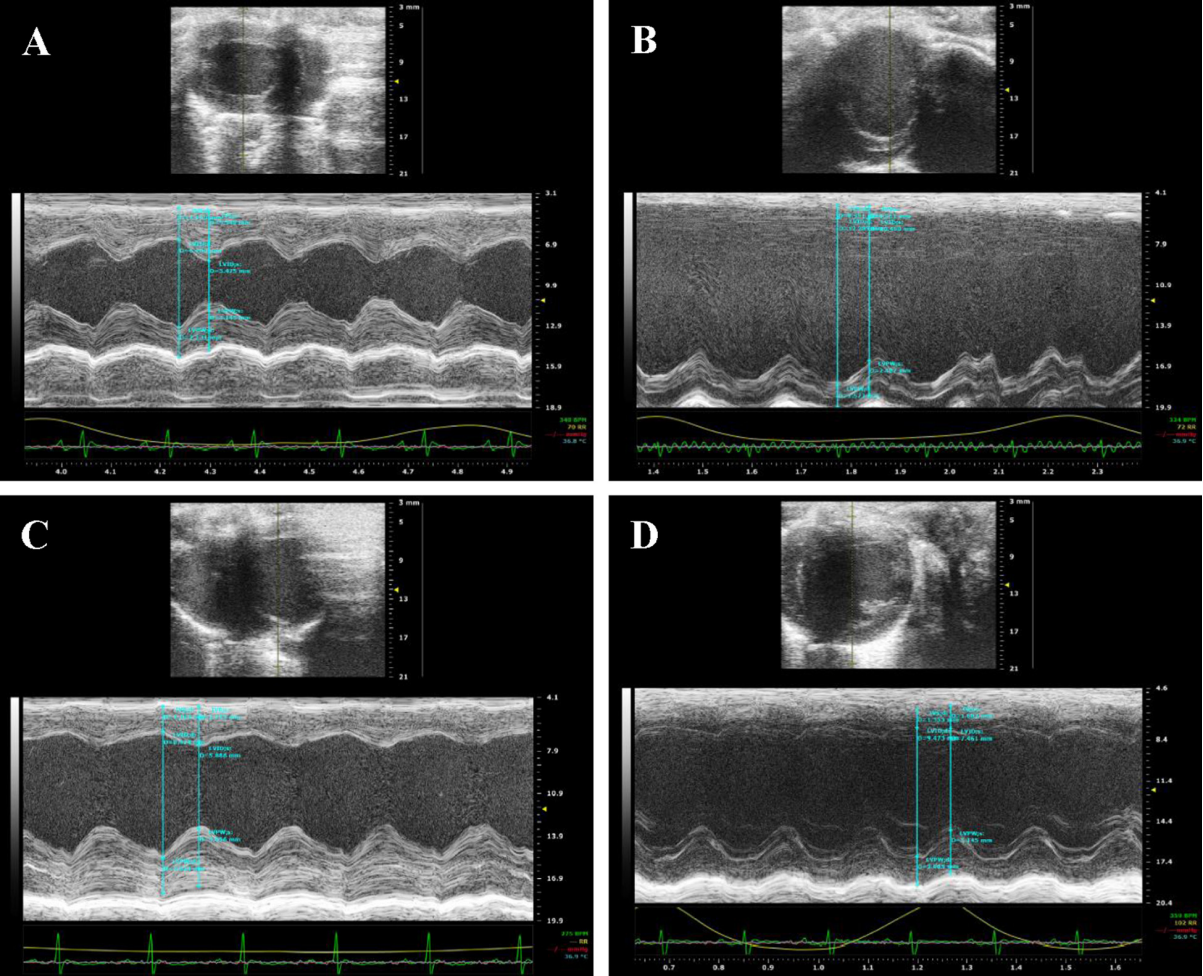
**Cardiac function detected by echocardiography. (A)** Normal cardiac function including LVEF and LVFS in sham-operated group. **(B)** Down-regulation of LVEF and LVFS in model group rats. **(C)** DQP can significantly up-regulate the EF and FS. **(D)** Positive Drug had no effects on the cardiac function.

Twenty eight days after surgery, echocardiography showed that EF and FS of rats in model group were significantly lower than that in the sham-operated group (Table 1, *P* < 0.01), indicating impaired cardiac function of rats in the model group. LVEDd, LVEDs and LV mass increased in the model group compared with those in the sham-operated group, suggesting the development of cardiac hypertrophy in this stage. After treatment with DQP for 28 days, the EF and FS were up-regulated by 26.55% and 38.20% respectively, compared with those in the model group. LVEDs was also improved significantly after treatment with DQP (Table 1, *P* < 0.01). In the positive control group, pravastatin showed no effect on cardiac function-related parameters compared with model group, as showed in Figure 1.

**Table 1:** **Cardiac function-related parameters in different groups** $\left(\overline{x}\pm \mathrm{s}\right)$

**Group**	**N**	**Sham**	**Model**	**Danqi pill**	**Positive drug**
**LV Mass (ug)**	12	647.29 ± 206.658^**^	936.29 ± 203.356	803.33 ± 213.294	872.65 ± 137.586
**IVS;d (cm)**	12	1.986 ± 0.316^**^	1.402 ± 0.378	1.532 ± 0.500	1.190 ± 0.380
**IVS;s(cm)**	12	2.915 ± 0.592 ^**^	1.658 ± 0.684	2.050 ± 0.886	1.476 ± 0.539
**LVEDd(cm)**	12	5.735 ± 0.656^**^	8.741 ± 1.2054	7.777 ± 1.869	9.107 ± 1.138
**LVED;s(cm)**	12	2.988 ± 0.829 ^**^	7.059 ± 1.535	5.746 ± 2.502^*^	7.538 ± 1.519
**LVPWd(mm)**	12	2.030 ± 0.441^**^	2.012 ± 0.389	1.953 ± 0.392	1.852 ± 0.361
**LVPWs(mm)**	12	2.758 ± 0.598^**^	2.792 ± 0.481	2.254 ± 0.618	2.560 ± 0.618
**EF (%)**	12	78.274 ± 10.514^**^	41.388 ± 17.238	52.375 ± 24.421^**^	34.823 ± 14.849
**FS(%)**	12	48.826 ± 10.557^**^	21.882 ± 10.552	30.241 ± 17.328^**^	17.905 ± 8.367

^*^ *P* < 0.05, ^**^ *P* < 0.01, Levels in the model group were used as reference to calculate *P* values.

### Effects of DQP on plasma HDL, LDL, TC and TG

Changes of plasma TC and TG levels are important indicators of lipid metabolism disorders [16]. In this study, plasma TG in the model group was up-regulated by 169.53% compared with that in the sham-operated group (Table 2, *P* < 0.01). Plasma TC was also increased by 16.30% in the model group but the difference was not statistically significant (*P* = 0.51). After treated with DQP and pravastatin, TG level was reduced by 66.67% and 39.54%, respectively (Table 2, *P* < 0.05). The level of TC showed no significant change in either DQP or pravastatin group.

**Table 2:** **Changes of plasma lipid indicators in different groups** $\left(\overline{x}\pm \mathrm{s}\right)$

**Group**	**N**	**TC(pg/ml)**	**TG(pg/ml)**	**HDL(pg/ml)**	**LDL(pg/ml)**
**Sham**	12	99.17 ± 24.30	6.40 ± 2.30^**^	56.20 ± 20.03^*^	2.86 ± 1.07^*^
**Model**	12	115.33 ± 23.63	17.25 ± 5.56	33.25 ± 7.14	5.67 ± 1.53
**Danqi pill**	12	119.30 ± 33.16	5.75 ± 4.43^**^	52.75 ± 1.2 6^*^	2.00 ± 1.69^**^
**Positive Drug**	12	105.56 ± 42.29	10.43 ± 5.32^*^	41.50 ± 12.71	4.13 ± 2.30^*^

^*^ *P* < 0.05, ^*^^*^ *P* < 0.01,Levels in the model group were used as reference to calculate *P* values.

HDL and LDL are important lipid transportation lipoproteins. The balance between them is important for the regulation of plasma level of lipid [17]. In this study, Plasma HDL level decreased by 40.84% in the model group compared with that in the sham-operated group (*P* = 0.018). After treatment with DQP for 28 days, an increase of HDL level was detected compared with the model group (*P* = 0.04), which almost returned to the level in sham-operated group. Pravastatin also up-regulated HDL level as was shown in Table 2. In addition, Plasma LDL increased by 98.25% in the model group (*P* = 0.031) compared with the sham-operated group and after treatment with DQP, the level was reduced by 64.73%. Pravastin could also reduce LDL level but to a less degree compared with DQP (Table 2).

### Effects of DQP on cardiac lipoprotein and HGMCR

To further investigate the mechanism by which DQP regulates lipid metabolism, we detected changes of key proteins in lipid metabolic pathway. Elisa results in this study showed that in model rats, plasma ApoA-I concentration was significantly lower than that in the sham-operated group (Table 3, *P* = 0.033), while Apo-B concentration increased by 141.67% (*P* = 0.003). After treatment with DQP, level of ApoA-I was increased and level of Apo-B was reduced significantly (*P* < 0.05). Pravastatin could also down-regulate Apo-B significantly and up-regulate ApoA-I to certain extent (Table 3). The levels of LP (a), another lipoprotein, were not significantly different in all of four groups.

**Table 3:** **Levels of Apolipoproteins and HMGCR in different groups** $\left(\overline{x} \pm \mathrm{s}\right)$

**Group**	**N**	**ApoA-I(pg/ml)**	**Apo-B(pg/ml)**	**LP(a) (pg/ml)**	**HMGCR(ng/ml)**
**Sham**	12	488.00 ± 3.04^*^	24.00 ± 13.02^**^	100.00 ± 12.35	2.86 ± 1.07^**^
**Model**	12	460.00 ± 9.33	58.00 ± 8.37	130.00 ± 42.43	5.67 ± 1.53
**Danqi pill**	12	482.86 ± 6.96^*^	16.67 ± 14.14^**^	102.00 ± 6.32	2.00 ± 1.69^**^
**Positive Drug**	12	471.11 ± 16.16	31.25 ± 17.27^**^	145.56 ± 87.91	1.13 ± 0.30^*^

^*^P < 0.05, ^*^^*^P < 0.01,Levels in the model group were used as reference to calculate *P* values.

**Figure 2:**
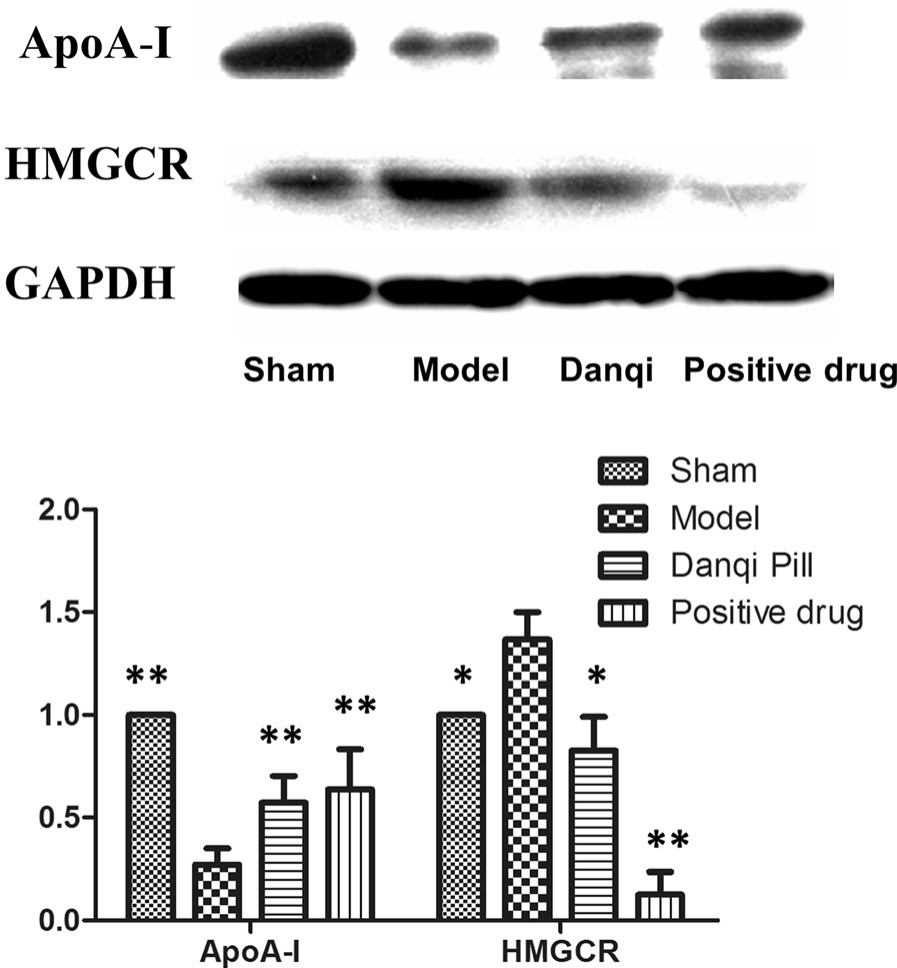
**The myocardial concentration of ApoA-I and HMGCR in different groups.**The results showed that in rats with CHD, plasma ApoA-I concentration was significantly lower than that in the sham-operated group. In DQP group, level of ApoA-I was increased significantly. Pravastatin could also up-regulate ApoA-I to certain extent. The HMGCoA expression showed the reverse pattern in different groups. ^*^ *P* < 0.05, ^*^^*^ *P* < 0.01. Levels in the model group were used as reference to calculate *P* values.

HMGCR is the most important target for the treatment of hyperlipidemia. Statins are a group of HMGCR inhibitors and verified to have efficacy for hyperlipidemia by large scale clinical experiments [18]. In rats with CHD, HMGCR was up-regulated by 98.25% compared with that in sham-operated group. As a specific HMGCR inhibitor, pravastatin reduced its level by 80.71% (*P* = 0.003). Meanwhile, DQP could also down-regulate HMGCR level significantly (64.73%, *P* = 0.017). The Elisa results were consistent with western blot results (Table 3 and Figure 2).

### Effects of DQP on FATP and CPTI proteins

**Figure 3:**
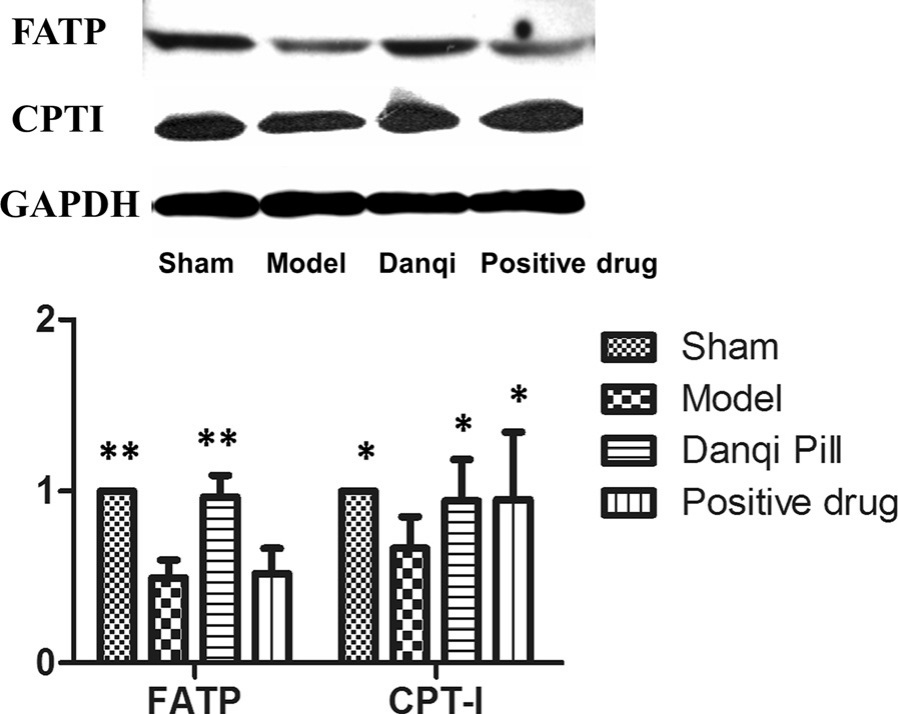
**The myocardial concentration of FATP and CPTI.** The results showed that FATP in the model group decreased compared with that in the sham-operated group. Level of Cardiac mitochondrial CPTI was also reduced. In DQP group, both the cardiac FATP and CPTI levels increased. Pravastatin increased the level of CPTI but had no significant effect on FATP. *P < 0.05, **P < 0.01. Levels in the model group were used as reference to calculate *P* values.

Lipid metabolism disorders in CHD are closely associated with defects in transportation and intake of fatty acids [19]. Western blot analysis of cardiac FATP showed that FATP level in the model group (0.49 ± 0.11) decreased by 50.61% compared with that in the sham-operated group (1.00 ± 0.00, *P* = 0.017). Level of cardiac CPTI was also reduced by 33.33% (0.67 ± 0.18, *P* = 0.031), suggesting that the ability of fatty acids intake by cardiac cells was impaired in rats with MI. After treatment with DQP, cardiac FATP and CPTI levels increased by 95.64% (0.97 ± 0.13) and 41.68% (0.94 ± 0.24) respectively, indicating that DQP could promote the intake of fatty acids by cardiac cells. Pravastatin increased the level of CPTI but had no significant effect on FATP (Figure 3).

### Effects of DQP on PPARα-RXR pathway regulation

**Figure 4:**
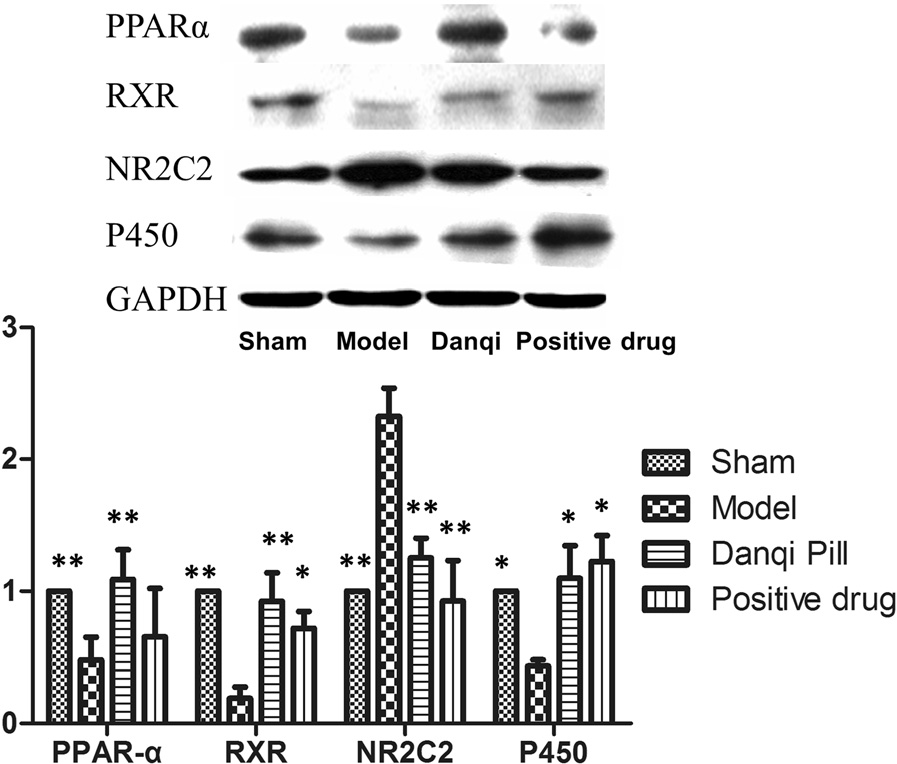
**The myocardial concentration of PPARα, RXR, NR2C2 and P450.** The results showed that the levels of PPARα and RXR decreased compared with sham-operated group. The level of NR2C2 increased significantly. A reduction level of P450 was observed in the model group. In DQP group, PPARα-RXR pathway was up-regulated, together with decreased level of NR2C2. P450 level was also increased. Pravastatin had similar efficacy on all these indicators as DQP, except for PPARα.*P < 0.05, **P < 0.01. Levels in the model group were used as reference to calculate *P* values.

PPARα-RXR pathway plays an important role in regulating fatty acids metabolism in cardiac cells [20,21]. In the model group of this study, the levels of PPARα and RXR decreased by 52.18% (0.48 ± 0.17) and 80.86% (0.19 ± 0.08) respectively compared with those in sham-operated group (*P* < 0.01). The level of NR2C2 (2.32 ± 0.216), which is a transcription inhibitor of PPARα-RXR pathway, increased significantly, further suppressing the activation of PPARα-RXR pathway (Figure 4, *P* < 0.001). P450 oxidase is a key enzyme in the oxidation process of fatty acids and a reduced level (0.43 ± 0.04) was observed in the model group (Figure 4, *P* = 0.044). After treatment with DQP, PPARα-RXR pathway was up-regulated, illustrated by an increased level of PPARα (1.09 ± 0.23) and RXR (0.92 ± 0.22) together with decreased level of NR2C2 (1.25 ± 0.15). P450 oxidase level (1.10 ± 0.25) was also increased in DQP group, thus promoting the degradation of lipids in cardiac cells [22,23]. Pravastatin had similar efficacy on all these indicators as DQP, except for PPARα.

## Discussion

According to lipid infiltration theory, lipid metabolism disorder is one of the major pathological mechanisms of myocardial ischemia induced by coronary heart disease [24,25]. However, few studies have been carried out to investigate if myocardial ischemia can cause lipid metabolism disorders and raise the plasma lipid directly. The verification of this causation would have important clinical implications: even if myocardial ischemia is not caused by high plasma lipid, the plasma lipid should also be monitored closely in case that the symptom was aggravated by lipid metabolism disorders. Our previous study indicated that myocardial ischemia could induce lipid metabolism disorders in swine ischemia model [13]. We observed that the levels of plasma ox-LDL, LDL and VLDL were up-regulated. Renin-angiotensin-aldosterone system was also activated together with lipid disorders [13]. However, the mechanism of lipid metabolism disorder hasn’t been illustrated so far. In this study, we aimed to study the mechanism of fatty acids metabolism disorder induced by pure myocardial ischemia in a comprehensive perspective of “circulation, intake and degradation” of fatty acids in myocardial ischemia models. We also aimed to explore the mechanism by which DQP regulates lipid metabolism in ischemia heart.

This study showed that in model rats with myocardial ischemia, the level of “good” lipoprotein HDL was down-regulated, while the level of “bad” LDL was increased by 98.3%. TG level was up-regulated, indicating that plasma lipid metabolism disorder was induced in our myocardial ischemia models. Under normal conditions, 70% of the energy supply of the normal cardiac cells comes from β oxidation of lipid [26]. Fatty acids in plasma are transported into cardiac cells by FATP on the membrane of cardiac cells. CPTI, a mitochondrial transmembrane enzyme, further transfers fatty acid into mitochondria for β-oxidation. Activation of CPTI is thought to be rate limiting for fatty acid entry into the mitochondria for oxidation [27,28]. Under the condition of cardiac ischemia, production of ATP is decreased. Tryglyceride is mobilized into plasma, degraded into glycerol and fatty acids and further oxidized to provide energy for cardiac cells [29]. Our study showed that in model rats with myocardial ischemia, the fatty acids transportation pathway was down-regulated, illustrated by decreased expression of FATP which uptakes free fatty acids from circulation. Besides, the expression of CPTI was significantly down-regulated, so the transportation of fatty acids into mitochondria for β-oxidation was also compromised. Furthermore, the transcription factors PPARα and RXR which could promote β oxidation of fatty acids, were also decreased in the myocardial ischemia model [20,30]. The degradation of fatty acids in cardiac cells was further down-regulated by increased expression of the metabolism pathway inhibitor NR2C2 [31]. Moreover, P450, which can utilize fatty acids as substrates to produce PPARα ligands, also decreased [32,33]. In summary, the deregulation of FATP, CPTI and PPARα could be the cause of fatty acids metabolism disorders in myocardial ischemia model.

DQP is a widely prescribed medicine for myocardial ischemia associated with coronary heart disease and has definitive efficacy [8]. It can prevent the aggregation of platelets and improve microcirculation [12]. However, the direct and comprehensive effects of DQP on fatty acids metabolism haven’t been reported yet. Previous study has suggested that Danshen, a component of DQP, could improve plasma lipid metabolism in hyperlipidemic rats [34]; Salvianolicacid IIA could regulate plasma lipid by improving the level of HDL in patients with coronary atherosclerosis[35]; Panax notoginseng saponins could improve lipid metabolism in aortic endothelial cells by inducing the expression of liver X receptor alpha (LXR-α) [36]. In this study, we investigated the effect of DQP on FATP-CPTI lipid metabolism pathway to explore its potential pharmacological mechanism.

Our study showed that DQP was able to regulate HDL and LDL levels, thus improving lipid metabolism in rats with MI. 28 days after treatment with DQP, FATP-CPTI pathway was activated and the level of PPARα and RXR increased significantly, almost back to normal level as that in the sham-operated group. PPARα–RXR are heterodimeric transcription factors that regulate lipid metabolism, by promoting β-oxidation of fatty acids and synthesis of Apo A-I and LPL, lowering TG and LDL levels and raising HDL level [21,30,37-39]. PPARα agonists, such as fibrates, have been used widely for the treatment of dyslipidemia [39]. DQP may activate PPARα in the similar way as fibrates. DQP also down-regulated the level of PPARα inhibitor NR2C2, further activating the transcriptional effect of PPARα [31]. Finally, DQP also can up-regulate the level of P450, further increasing the activity of PPARα.

**Figure 5:**
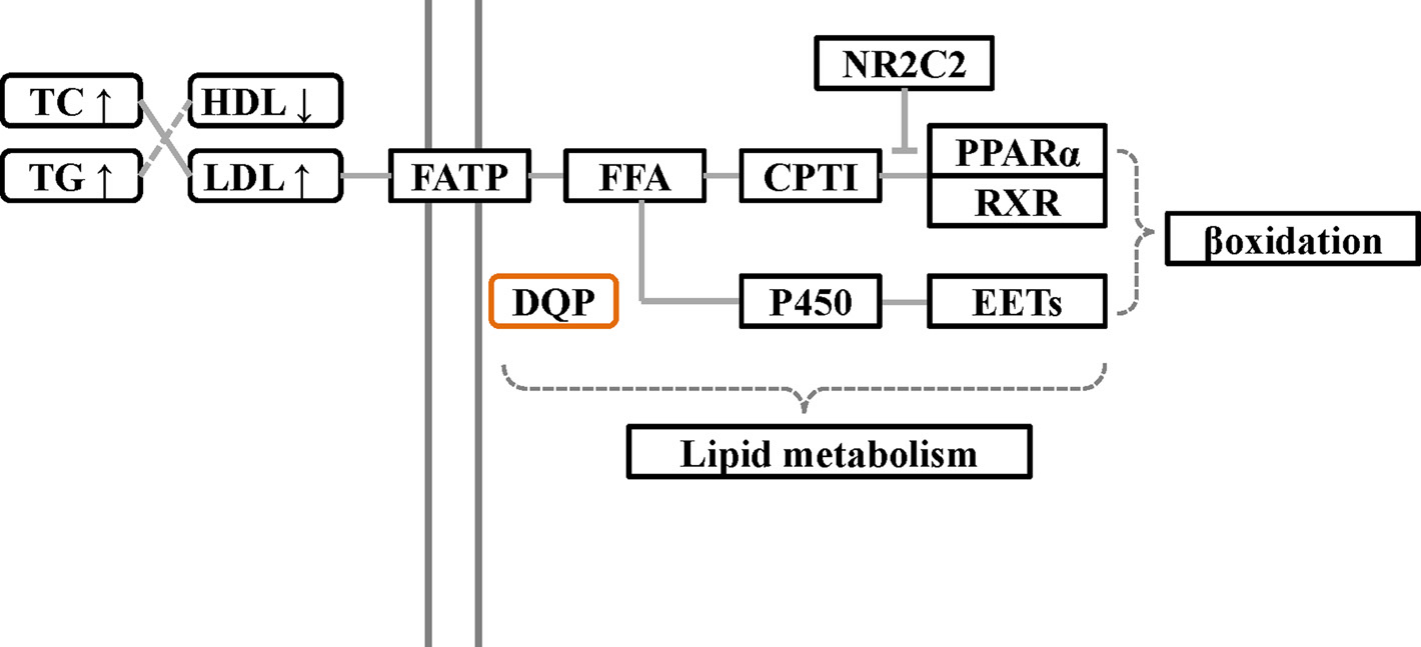
**The potential mechanism of DQP regulating the lipid disorder induced by myocardial ischemia.**

Those results suggest that DQP could promote the intake and breakdown of free fatty acids by effectively activating FATP-CPTI pathway and relieving the suppression effect of NR2C2 on PPARα pathway (Figure 5). The lipid metabolism disorder induced by myocardial ischemia in model rats could be greatly improved by DQP administration.

Pravastatin was used as positive control in this study. Statins are used as the first-line drugs for the treatment of dyslipidemia, by inhibiting the enzyme HMG-CoA reductase [30]. The results of this study showed that DQP could regulate lipid metabolism as effectively as pravastatin. Furthermore, DQP was able to improve heart function and up-regulate ejection fraction by increasing the cardiac diastolic volume. So our study reveals that DQP would be an ideal alternative drug for the treatment of dyslipidemia which is induced by myocardial ischemia

## Conclusions

In conclusion, we not only explored the pathological mechanism of lipid metabolism disorder induced by myocardial ischemia in MI rats model, but also investigated the regulatory efficacy of DQP on lipid metabolism. This study provides new insights into ways for clinical management of lipid metabolic disorders induced by MI.

### Limitation

There are a few limitations in our study. Firstly, even though the dyslipidemia model induced by myocardial ischemia has been created in different species such as swine and rats, more experiments are still needed to further validate this result. In particular, more clinical data needs to be analyzed to support the result. Secondly, we focused on lipid metabolism disorders induced by myocardial ischemia only in myocardial tissues and plasma. We didn’t analyze lipid metabolism in other tissues such as liver and intestines. More studies will be carried out to comprehensively analyze lipid disorders induced by myocardial ischemia in other tissues.
